# The paradox of mentally ill psychiatrists: a punch to the mental-health related myths and prejudices around the profession of psychiatry

**DOI:** 10.1192/j.eurpsy.2025.2009

**Published:** 2025-08-26

**Authors:** G. N. Porfyri, V. Tarantili

**Affiliations:** 1 National and Kapodistrian University of Athens, Athens; 2 General Hospital of Argos, Argos, Greece

## Abstract

**Introduction:**

As reported by studies and against the general conception, psychiatrists are not immune to the mental health challenges. More specifically, -more than any other medical specialty- they are at high-risk for burnout, depression, even suicide. It is a striking fact that according to research, in a medical centre which was exclusively designated for healthcare professionals, 89% of psychiatrists struggled with mental health issues; while 8% faced substance abuse problems and only 2% complained for corporal matters. Furthermore, when suffering from depression or any other mental health disorder, psychiatrists fear that their personal data will leak among colleagues, that they will be unable to professionally evolve or that they will be shamingly judged, avoiding to seek medical help.

**Objectives:**

To explore the risk factors of poor mental health among psychiatrists and to highlight interventions to reduce the mental-health related stigma in this particular category.

**Methods:**

A review of 39 articles -from 2010 to 2024- on PubMed and Google Scholar regarding mental health problems among psychiatrists.

**Results:**

Numerous risk factors of psychiatrists’ poor mental health have been identified, such as:

Female gender,

Younger age,

Race minority,

Prior mental health problems,

Residency or early career stage,

Working in non academic, multidisciplinary, inpatient, community, and government settings,

>50 h of work per week and/or more than 20 h of direct clinical face time per week,

Insufficient support from relatives, workplace, and colleagues,

Lack of supervision,

Lack of control over schedule,

Experiencing loneliness,

Experiencing work unsatisfaction,

Experiencing traumatic events such as patient’s suicide or receiving threats.

**Conclusions:**

Action against mental health-related stigma among psychiatrists needs to be taken, such as destigmatizing campaigns designated to remind that psychiatrists -as normal human beings- can suffer from mental health problems in the same way a cardiologist could have a cardiac attack. Apart from destigmatizing mental health issues among psychiatrists, legislation in every country needs to be changed in order to protect psychiatrists from work overload, while security of mental health professionals must be maintained in every clinical setting preventing the reception of threats or even physical abuse. Additionally, female psychiatrists should be institutionally empowered through mentorship programs, sponsorship support, responsive caregiving programs, and innovational directions to manage implicit and explicit prejudices, sexual harassment, and remuneration discrepancies.

**Disclosure of Interest:**

None Declared

